# Analysis of Balance, Rapidity, Force and Reaction Times of Soccer Players at Different Levels of Competition

**DOI:** 10.1371/journal.pone.0077264

**Published:** 2013-10-10

**Authors:** Leonardo Ricotti, Jacopo Rigosa, Alberto Niosi, Arianna Menciassi

**Affiliations:** The BioRobotics Institute, Scuola Superiore Sant’Anna, Pontedera (Pisa), Italy; University of Pittsburgh, United States of America

## Abstract

In the present study we analyzed 12 physical parameters, namely force, static and dynamic balance (both quantified by means of 4 parameters each), rapidity, visual reaction times and acoustic reaction times, over 185 subjects. 170 of them played soccer in teams enrolled in all the ten different Italian soccer leagues. Results show that 6 parameters (out of the 12 analyzed) permit to identify and discriminate top-level players, among those showing the same training frequency. The other parameters are strictly related to training frequency or do not discriminate among players or control subjects (non-athletes), such as visual and acoustic reaction times. Principal component analysis permits to identify 4 clusters of subjects with similar performances, thus representing a useful instrument to characterize the overall ability of players in terms of athletic characteristics, on the basis of their location on the principal component parameters plane.

## Introduction

This paper aims at reporting soccer players’ performance in terms of maximum vertical jump height (to determine maximal leg strength), contact time (to assess acyclic rapidity, or quickness), static balance, dynamic balance and visual and acoustic reaction times. This analysis was conducted by taking into account athletes playing in all the ten categories (four professional and six non professional) of the Italian soccer championship, and by analyzing at least fifteen athletes for each category. A control group, including subjects which did not played soccer, or other sports, was also included. This study also aims at demonstrating that a subgroup of the above mentioned characteristics permits to discriminate top-level athletes, among those showing the same training frequency. 

Soccer is the most popular team sport worldwide [1], with more than 250 million active players [2]. In general, the formation of a mature athlete necessarily entails the expression of a series of athletic characteristics in a proper and timely manner. Children and adolescents, in fact, are subjected to a maturation process that is not linear, but characterized by “developmental spikes”, affecting their capability to learn specific motor skills at certain ages [3].

Multidimensional performance analysis recently emerged as an effective tool to discriminate talented athletes. Elferink-Gemser and colleagues identified anthropometric, technical, tactical and physiological characteristics that could be able to predict future elite hockey players [4]. More recently, Rikberg and Raudepp measured anthropometric, physical, technical and cognitive characteristics of junior volleyball players, with the aim of discriminating their overall ability [5]. Despite the wide scientific interest in this field and the large number of studies performed, literature lacks of a detailed multiparametric study reporting data on force, rapidity, static and dynamic balance and reaction times of soccer players at different levels of competition. 

Besides technical and tactical skills, which are of primary importance in soccer, physical characteristics are actually crucial to discriminate talented from non-talented soccer players. Endurance and (partly) force are much more affected by training frequency and quality in comparison with other characteristics, such as rapidity, balance and reaction times [3]. 

During a game, professional soccer players perform about 50 turns, comprising sustained forceful contractions to maintain balance and control of the ball against defensive pressure. Hence, force and power expression is an important characteristic of high-level soccer players. Power is, in turn, heavily dependent on maximal leg strength [6].

Acyclic rapidity (or quickness) is the ability to perform a single (non repeated) movement in the shortest time and it is a crucial skill in soccer. The analysis of contact times is an effective means to evaluate athletes’ acyclic rapidity and it was recently used to quantify the performance of professional soccer players during lateral plyometric exercises [7].

Coordinative abilities (dexterity) rely on the movement control and regulation processes: they are of crucial importance in many sports, including soccer, as they allow athletes to easily control their motor actions. Moreover, they permit to learn complex movements in a relatively rapid way. One of the main components of coordinative abilities is balance. Postural control (or balance) can be defined statically as the ability to maintain a base of support with minimal movement (thus minimizing body sway), and dynamically as the ability to perform a task while maintaining a stable position [8]. Balance is influenced by a number of factors, such as sensory information (from somatosensory, visual and vestibular systems), joint range of motion and strength [9,10,11] and it is responsible for the correct execution of complex sport tasks.

Static and dynamic balance performances are often assessed by means of center of pressure (COP) recordings, by using sensorized platforms. Even if COP differs from center of mass (COM), it has been demonstrated that COM trajectory can be computed from the COP one [12], thus justifying COP measurements (relatively easy to obtain) for the assessment of body sway [13]. The relationship between balance ability and athletic performance has been recently deeply reviewed by Hrysomallis, who highlighted the insights achieved in the last two decades about postural control related to athletes’ performance in various sports [14]. Static and dynamic balance was compared between athletes performing different sports, finding that dancers show better static balance than soccer players [15,16], while gymnasts and soccer players do not differ in terms of both static and dynamic balance, also showing superior postural control in comparison with basketball players [17].

Paillard and Noe analyzed the importance of visual information in soccer players according to their level of competition. They found that professional players are less dependent on vision to control their posture in comparison with non professional athletes, thus suggesting that professional players are able to dedicate vision to treat the information emanating from the match [18]. Similar findings have been recently reported by Ben Moussa and colleagues, by comparing the contribution of vision on postural maintenance in professional and amateur soccer players [19].

Reaction times depend on motor nerve conduction velocity and are commonly divided between auditory reaction times (ART) and visual reaction times (VRT). It has been demonstrated that ART are less important than VRT in soccer, since it is essentially a visual game [20,21].

The findings reported in this paper clarify which characteristics are more suitable to discriminate high-level from lower-level soccer players, also between athletes showing the same training frequency. Furthermore, a principal component analysis allows to identify clusters of players with similar performances, thus permitting to resume their characteristics by means of only two parameters, accounting for a significant percentage of data variance.

## Materials and Methods

### Subjects

Ten groups of male soccer players (at least 15 subjects per each group) were involved in the study. Each group represented a different category of the Italian soccer championship, from the highest to the lowest level. A control group was also included in the study: to this aim, 15 subjects without any soccer or other sports experience were analyzed. The subjects involved showed an overall age of 23.3. ± 4.9 years, a height of 179.0 ± 5.7 cm and a weight of 74.7 ± 7.8 kg. Athletes playing in professional and non-professional categories obviously differed in terms of training frequency, while athletes playing in the four professional categories were all characterized by the same training frequency (Table 1).

**Table 1:** Group labels and number of subjects involved in the study.

**Group label**	**Category Italian name**	**Level**	**Weekly training frequency**	**No. of athletes analyzed**	**Age (years)**	**Height (cm)**	**Weight (kg)**
A	Serie A	*Professional*	5-7	15	26.2 ± 3.7	181.9 ± 6.9	79.4 ± 4.7
B	Serie B	*Professional*	5-7	16	23.4 ± 5.1	182.6 ± 2.4	78.1 ± 4.0
C	Lega Pro - 1a Divisione	*Professional*	5-7	15	21.4 ± 1.7	182.7 ± 3.5	78.9 ± 4.4
D	Lega Pro - 2a Divisione	*Professional*	5-7	17	25.3 ± 4.1	180.6 ± 5.5	77.8 ± 7.9
E	Serie D	*Non professional*	4-5	17	19.9 ± 3.7	180.0 ± 4.2	72.5 ± 6.6
F	Eccellenza	*Non professional*	4	23	22.3 ± 5.8	178.3 ± 4.7	74.1 ± 7.5
G	Promozione	*Non professional*	3	18	21.2 ± 3.1	177.3 ± 5.4	70.4 ± 4.5
H	Prima Categoria	*Non professional*	3	16	22.6 ± 4.4	174.4 ± 6.0	68.5 ± 5.8
I	Seconda Categoria	*Non professional*	2	17	24.8 ± 5.5	174.8 ± 7.1	71.1 ± 9.4
L	Terza Categoria	*Non professional*	2	16	24.4 ± 4.7	176.9 ± 4.6	73.5 ± 6.1
X	-	*Control group*	-	15	27.3 ± 5.2	177.1 ± 4.4	73.7 ± 8.8
			**TOT**	**185**	**23.3 ± 4.9**	**179.0 ± 5.7**	**74.7 ± 7.8**

Level (professional or non professional) and typical weekly training frequencies for the different soccer categories are also reported, as well as age, height and weight of the subjects involved in the study. Data are reported as mean value ± standard deviation.

A brief interview was carried out before starting the experiments. To be included in the study, subjects should not be injured, nor recovering from ankle, knee, hip or other known injuries. Furthermore, goalkeepers were excluded, as well as subjects that had performed dance, judo or other martial arts for more than six months in their life. Experiments were conducted at the beginning of the competitive season. All the subjects signed an informed consent as required by the Declaration of Helsinki. The study was approved by the local ethics committee of Scuola Superiore Sant’Anna.

### Tests and instruments

**Figure 1:**
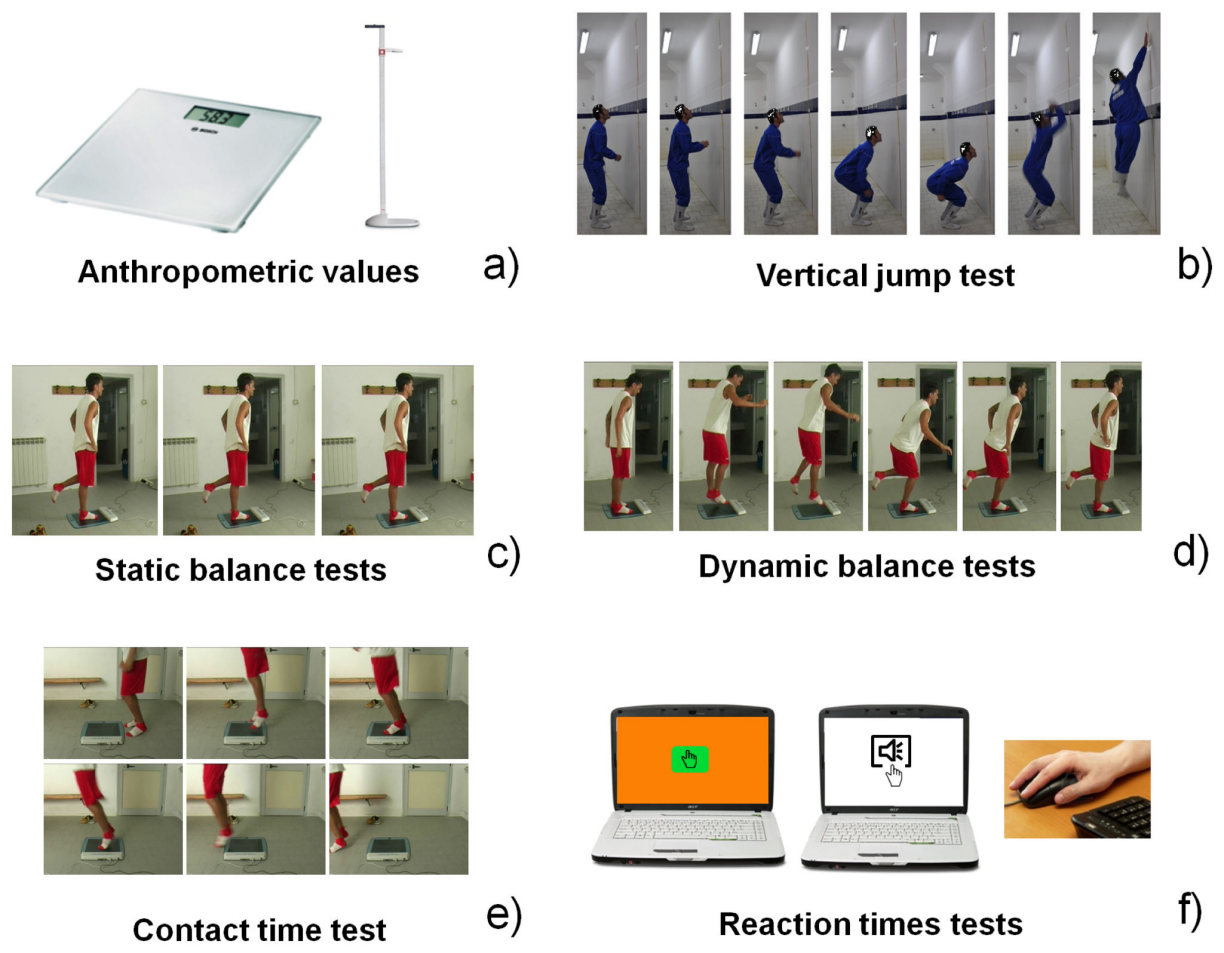
Overall view of the tests carried out on the subjects involved in the study. a) measurement of anthropometric values by means of dedicated tools; b) assessment of maximum vertical jump height; c,d) static and dynamic balance tests by means of a force platform; e) assessment of subject’s rapidity by means of contact time measurements; f) assessment of visual and acoustic reaction times by means of a dedicated software.

First, the anthropometric data of each athlete were registered (Figure 1a). Weight was assessed by means of a standard digital balance (Seca, max 200 kg), while height was measured by using a wall-mounted stadiometer (Siber Hegner). Then, athletes’ force was assessed by means of a vertical jump test (Figure 1b). A wall-mounted graduated tape allowed to record vertical maximum jump height. Both static and dynamic balance (Figure 1c,d) were assessed by means of a force platform (WinPosture, Imago snc) that recorded the displacements of the centre of foot pressure (COP) with 1.56 sensors/cm^2^ and recording in “postural acquisition mode” at 100 Hz. The same platform was used to record contact times during rapidity tests (Figure 1e), by using a “dynamic acquisition mode” at 150 Hz. Finally, visual and acoustic reaction times were recorded by using a personal computer (PC) equipped with a dedicated software (Reaction Times, freely available on the net, Figure 1f).

### Protocol

The tests were conducted in a discrete room free from external distractions and approximately at the same hour, to avoid the possibility of obtaining discrepancies between subjects’ performance (especially concerning balance) due to difference in time of day [22].

After the interview, aiming at identifying and selecting the subjects to involve in the study, soccer players’ age, height and weight were recorded. Then, a brief warm-up (5 min running) was performed. Before vertical jump tests, the total body length of the subject was measured, by asking him to touch the graduated tape with both hands at the highest point possible, without raising the heels from the floor. This value was registered as L_t_ (total length). During vertical jump tests, subjects started from a standing position and performed a crouching action, immediately followed by a jump for maximal height. Each subject performed the test three times, with two minutes of rest for complete recovery between jumps. The hands were left free to move while jumping and the athlete was asked to touch a point on the graduated tape at the maximum height he could reach. The highest value obtained was recorded and named H_j_ (height reached with the jump). Athlete’s force performance was quantified as follows:

$$\Delta L=H_{j}-L_{t}$$ (1)

Vertical jump tests based on three repetitions for each athlete have been demonstrated to represent effective means to measure bilateral leg force, to discriminate between individuals of different performance levels, and to detect training-induced changes to performance [23].

Static balance tests were characterized by unipedal standing postures on both dominant and non-dominant legs. The dominant leg was identified before starting the test as the leg the subject preferentially used to kick the ball. First, the dominant leg was tested: the subject was asked to take position on the force platform, with the standing foot in the centre of the platform, looking at a fixed visual target on the wall (positioned at a distance of 3 m), to raise the non-dominant leg, to keep it flexed 90° at the knee, and to maintain a static position as long as possible for the entire duration of the test (20 s, during which COP displacements were recorded), by keeping both hands on his hips (Movie S1). After this, the subject was allowed to rest for 2 min and then asked to repeat the test, this time raising the dominant leg. A static balance test was repeated when the raised foot touched the surface or the subject moved away the hands from his hips during the test. In order to quantify static balance performances, two parameters were taken into account: COP length (the “travelling distance”, in mm, of COP displacement during the 20 s test) and COP area (the area of the confidence ellipse that encloses 95% of the COP points during the 20 s test). 

Dynamic balance tests were also performed for both dominant and non-dominant legs. In this case the subject took position on the force platform with the feet axes parallel to the main axis of the force platform and keeping a distance of 25 cm between the feet.

Then, COP was recorded for 20 s after a small jump (~ 20 cm) landing with only one foot. Once landed, the subject was asked to recover as soon as possible the equilibrium and to stabilize in the unipedal stance, also keeping his hands on the hips and looking at a fixed visual target on the wall, positioned at a distance of 3 m (Movie S1). A rest of 2 minutes was allowed between the two tests. A dynamic balance test was repeated if, after landing, the raised foot touched the surface or the subject moved away the hands from his hips. To quantify dynamic balance performance we considered two parameters, corresponding to COP length respectively 3 and 10 s after subject’s landing.

COP-based postural sway measurements, utilized in this study to quantify both static and dynamic balance, have been demonstrated to be highly reliable by previous literature works [24],[25],[26].

To assess rapidity, the subject was positioned laterally respect to the force platform and asked to perform a small jump on it, setting the instrument on “dynamic” recording modality. Once landed, the subject should jump again as soon as possible leaving the platform area, in order to minimize the contact time on the sensorized surface (Movie S2). Each subject performed the test three times, with 30 s of rest for complete recovery between jumps. The hands were left free to move while jumping. The smallest contact time obtained was recorded and considered as the subject’s rapidity performance. Contact time measurements are considered effective and reliable means to assess subject’s both cyclic and acyclic rapidity, as reported by previous literature examples [3],[27].

For both visual and acoustic reaction time tests the subject was asked to sit on a chair in front of a PC equipped with a dedicated software, with the dominant hand grabbing a mouse and ready to click. The visual test consisted in a series of six visual stimuli appearing at random time intervals on the PC screen: the subject was asked to click as rapidly as possible once he saw the visual stimulus. The acoustic test consisted in a series of six acoustic stimuli generated by the software at random time intervals: the subject was asked to click as rapidly as possible once he heard the acoustic stimulus. For both tests, 5 consecutive trials were performed, with short resting periods between the tests. For each trial, the first two attempts (not recorded) were needed by the subject to familiarize with the procedure and to reach the highest attention level (the first values were often much higher than the following ones, probably due to a drop of attention after the resting periods). Therefore, only the four final values of each trial (20 values in total for each test) were considered and used for the mean value calculation. Such value represented the visual/acoustic reaction time of the subject. This procedure and this number of trials and repetitions have been demonstrated to be sufficiently reliable for the determination of subjects’ reaction times [28].

### Statistical analyses

The statistical analyses of the collected data were based on the null hypothesis, which is founded on the assumption that there was not a significant difference between the measured values, for the different tests, concerning the soccer players and the control subjects involved in the study. A one-way ANOVA, where each group represented a different category, was used to assess the existence of any violation of the null hypothesis assumption.

For each test, a series of coupled t-tests was then performed, by comparing each category with all the other ones. The results were plotted as a matrix of colored squares (with each color corresponding to a specific p value) which made easier the identification of clusters of athletes with similar performance. The significance level was set at 0.05.

Since considering a set of statistical inferences simultaneously causes more likely type I errors, i.e. incorrect rejections of correct null hypotheses [29], post-hoc multiple comparisons of the one way ANOVA(s) were also considered. As a consequence, a stronger level of evidence should be observed in the phenomenon to be "significant". The Bonferroni correction is considered to be the most conservative method to control the familywise error rate (i.e. the probability of making false discoveries) in a multiple comparisons problem. Briefly, assuming *m* as the number of groups, in a multiple comparisons problem we need to determine *m* confidence intervals (CIs) with an overall confidence level of *1* - α, where α is the significance level. The Bonferroni correction adjusts each individual CI according to the following equation:

$$CI=1-\frac{\alpha }{m}$$ (2)

Based on the above considerations, we completed our statistical analysis by reporting, for each test, the average value and 95% of the CI (calculated by means of Bonferroni correction) of each category.

For further meta-analyses of the obtained results we also reported an effect size, namely the Cohen’s coefficient f^2^, defined as:

$$f^{2}=\frac{R^{2}}{\left(1-R^{2}\right)}$$ (3)

where R^2^ is the squared multiple correlation.

Due to the considerably different variances that characterized the collected data, we opted for a non-parametric ANOVA coupled with a resampling method, in particular a repeated random sub-sampling validation. For each test, we performed 100 non-parametric ANOVAs on sub-groups composed of 7 subjects, randomly selected within each experimental group. The distribution of the correspondent obtained p-values was then reported, for each test.

In correspondence to the non-parametric ANOVAs coupled with a resampling method, we calculated the Cohen’s coefficients accordingly. In this case, we considered the expression of f^2^ in the case of a balanced design (equivalent sample sizes across groups), namely:

$$f^{2}=\frac{SS(\mu_{1},\mu_{2},...,\mu_{K})}{K\times \sigma }$$ (4)

where SS is the sum of squares manipulation in ANOVA, μ_i_ is the population mean within the i^th^ group of the total K groups and σ includes the equivalent population standard deviations within each group. f^2^ was considered “small” if around 0.02, “medium” if around 0.15 and “large” if around 0.35.

Finally, we performed a principal component analysis (PCA), by converting a subclass of the observations into linearly uncorrelated variables (the principal components, PCs). More specifically, the observations that we included in the PCA were those referring to force, static balance (COP length values for both dominant and non dominant limbs and COP area values, only concerning non dominant limb), dynamic balance (COP length values 10 s after landing, only for non dominant limb) and rapidity tests. This choice was determined by the experimental results (described in the next section), which highlighted significant differences between athletes showing the same training frequencies (even if playing in different categories) only for the mentioned parameters. PCA is known to be a powerful instrument for data reduction. This is useful when large amount of data may be approximated by a moderately complex model structure [30]. In our specific case, PCA was useful to investigate the topological distribution of the subjects on the plane identified by the first two principal components (accounting ~ 70% of the data variance), with the aim of scattering all the subjects and to identify distinct classes of athletes, grouped according to their performances in the different executed tests. In this way, it was possible to resume such performances by means of only two parameters (the PCs), which were linear combinations of the mentioned tests outcomes. Data analyses were all performed by means of the software MATLAB (Mathwork Inc., MA), by using both existing and *ad hoc*-developed routines.

Experimental data and MATLAB codes used for the described analyses are available as on-line supporting files (Files S1, S2, S3, S4 and S5).

## Results and Discussion

### Force performance

**Figure 2:**
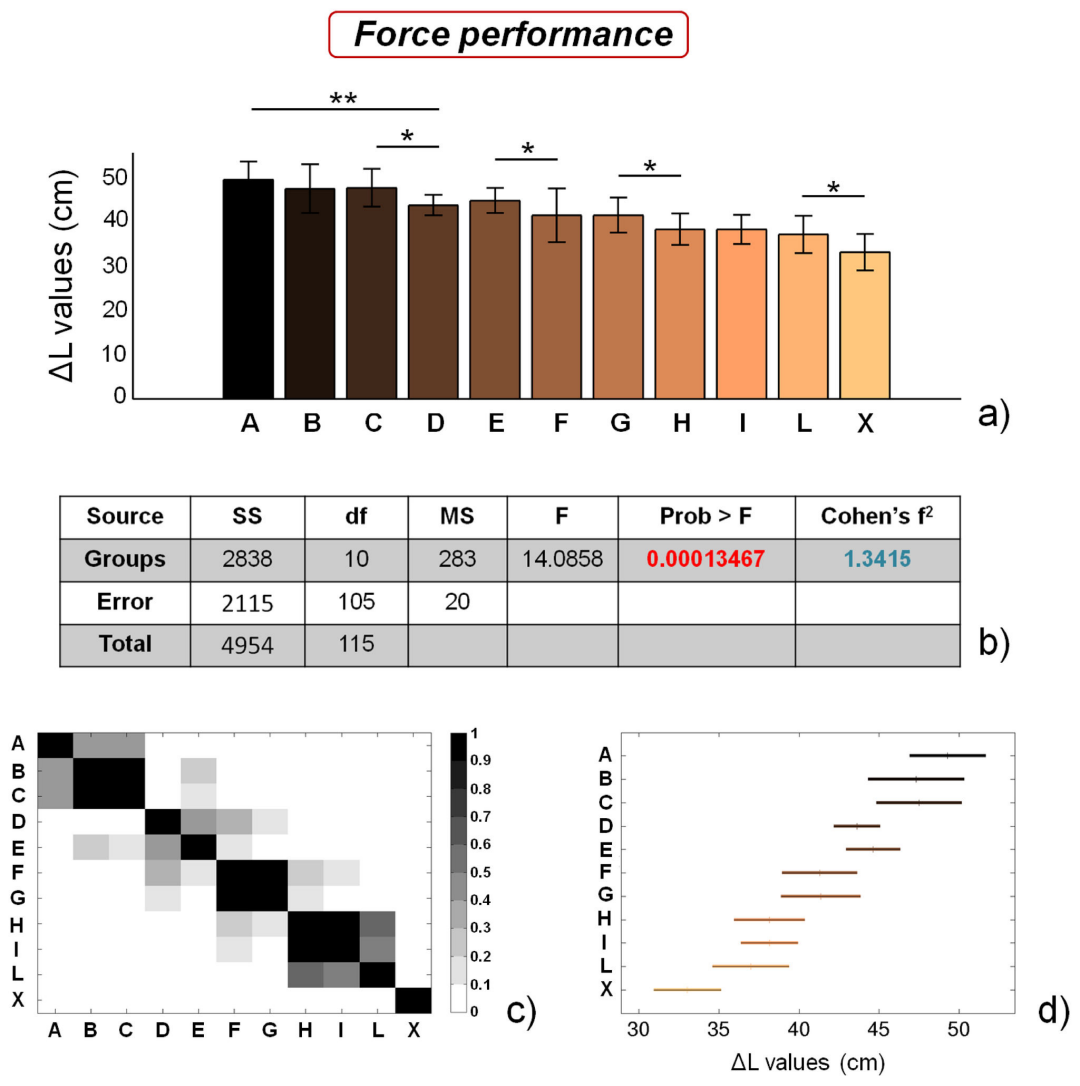
Force performance results. a) ΔL values for the different analyzed groups (average value ± standard deviation), from A to X. *=p<0.05, **=p<0.01; b) results of the analysis of variance (ANOVA) and effect size (Cohen’s f^2^) calculation; c) matrix reporting the p values for the coupled t-tests between the different groups; d) multiple comparison plot (average values ± 95% of CI, with Bonferroni adjustement).

Figure 2a reports the ΔL values, calculated as described in (1), for soccer players playing in the categories from A to L and for the control group (X). The ANOVA results, reported in Figure 2b, suggest that the groups are characterized by significantly different force performances (p value, highlighted in red, is much smaller than 0.01), a conclusion that is further confirmed by the Cohen’s f^2^ effect size, which is much larger than 0.35. Figure 2c shows a graphical representation of the p values of single statistical comparisons between groups, while Figure 2d shows a plot of the ΔL average values ± 95% of CI for the different groups, calculated by applying the Bonferroni correction.

Isokinetic strength and anaerobic power have been analyzed in the past years for elite, sub-elite and amateur soccer players. Results revealed that professional players differ from amateurs in terms of knee flexor muscle strength [31]. Maximal isometric force, force-time curve characteristics and vertical jump were also measured in young soccer players at different competition levels, finding that elite athletes expressed significantly higher strength characteristics in comparison with sub-elite and recreational counterparts [32]. More recent studies aimed at comparing strength-related parameters in young or adult soccer players at different levels of competition, focusing on maximal strength [33], full squat power output [34], jumping ability [35] and even specific muscle characteristics by means of tensiomyography [36].

In our case, ΔL values decrease almost linearly between A and L categories, without defining specific clusters of athletes with similar force performances. In addition, results show that control subjects show force performances significantly lower than those of soccer players playing in the L category (p<0.05). The obtained results confirm previous literature findings, reporting that soccer players at different levels of competition show different force performance. However, they also highlight that athletes showing the same training frequency (groups C and D, but also groups G and H) show different force performances. Force is generally strongly dependent on training frequency [3,31], but it is known that it is also partly related to athlete’s intrinsic factors, such as muscle fibre composition, neuromuscular control, etc [37,38]. Such training-unrelated factors would therefore explain the significant differences that we found in force performance between soccer players showing the same training frequency.

### Static balance performance

**Figure 3:**
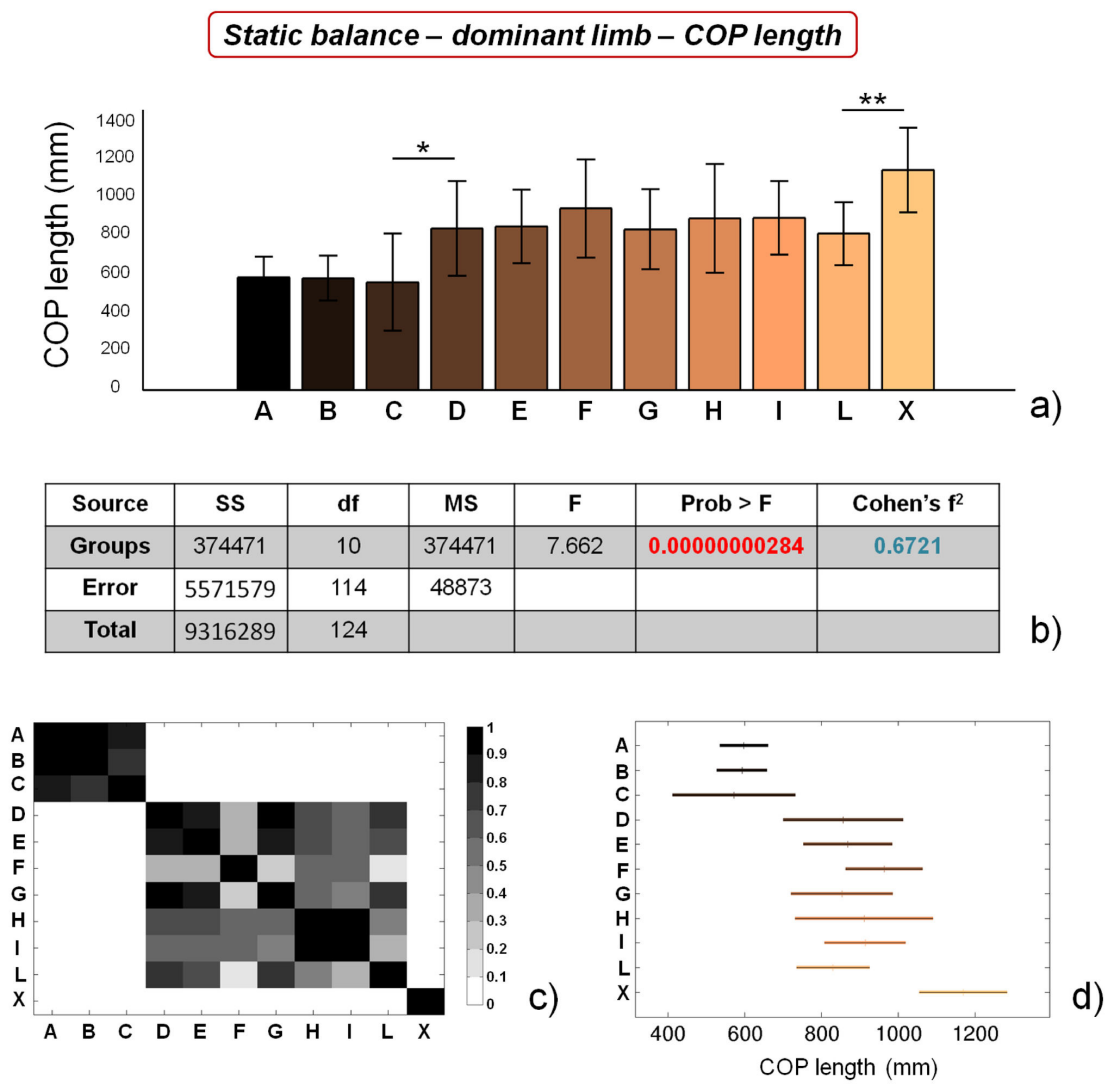
Results of static balance tests, evaluated by means of COP length values, on dominant limb. a) COP length values for the different analyzed groups (average value ± standard deviation), from A to X. *=p<0.05, **=p<0.01; b) results of the analysis of variance (ANOVA) and effect size (Cohen’s f^2^) calculation; c) matrix reporting the p values for the coupled t-tests between the different groups; d) multiple comparison plot (average values ± 95% of CI, with Bonferroni adjustement).

**Figure 4:**
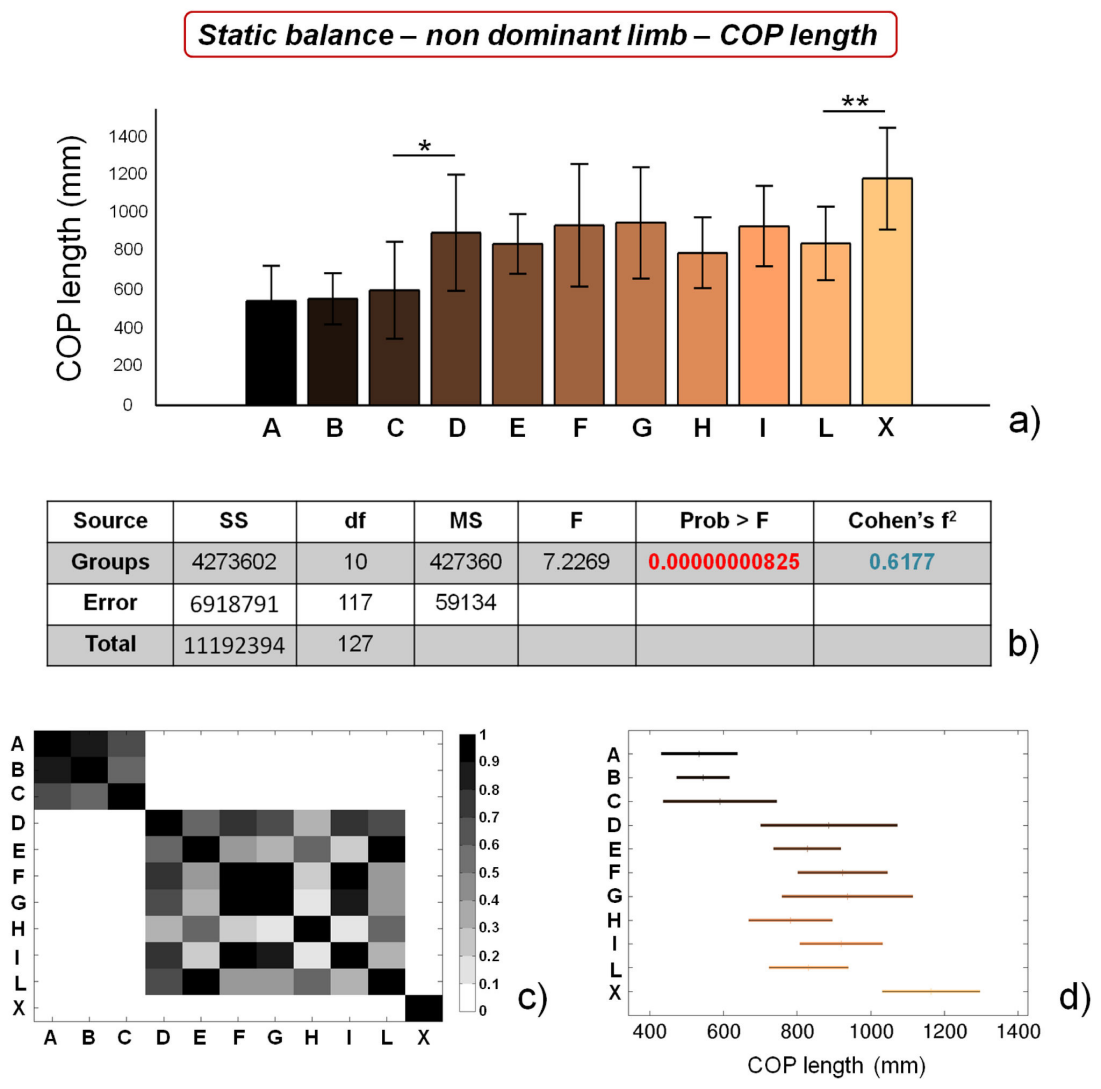
Results of static balance tests, evaluated by means of COP length values, on non dominant limb. a) COP length values for the different analyzed groups (average value ± standard deviation), from A to X. *=p<0.05, **=p<0.01; b) results of the analysis of variance (ANOVA) and effect size (Cohen’s f^2^) calculation; c) matrix reporting the p values for the coupled t-tests between the different groups; d) multiple comparison plot (average values ± 95% of CI, with Bonferroni adjustement).

COP length values for the different categories, concerning static balance tests on dominant limb, and the corresponding statistical analyses, are reported in Figure 3. Two macro-groups of soccer players with similar performance can be identified, the former constituted by athletes from A to C categories, the latter constituted by athletes from D to L categories. Control subjects significantly differ (p<0.01) from athletes playing in the L category. ANOVA analysis reveals significant differences between the groups, while Cohen’s f^2^ shows a considerably high value. Similar results were obtained by analysing COP length values concerning static balance tests on non dominant limb (Figure 4).

**Figure 5:**
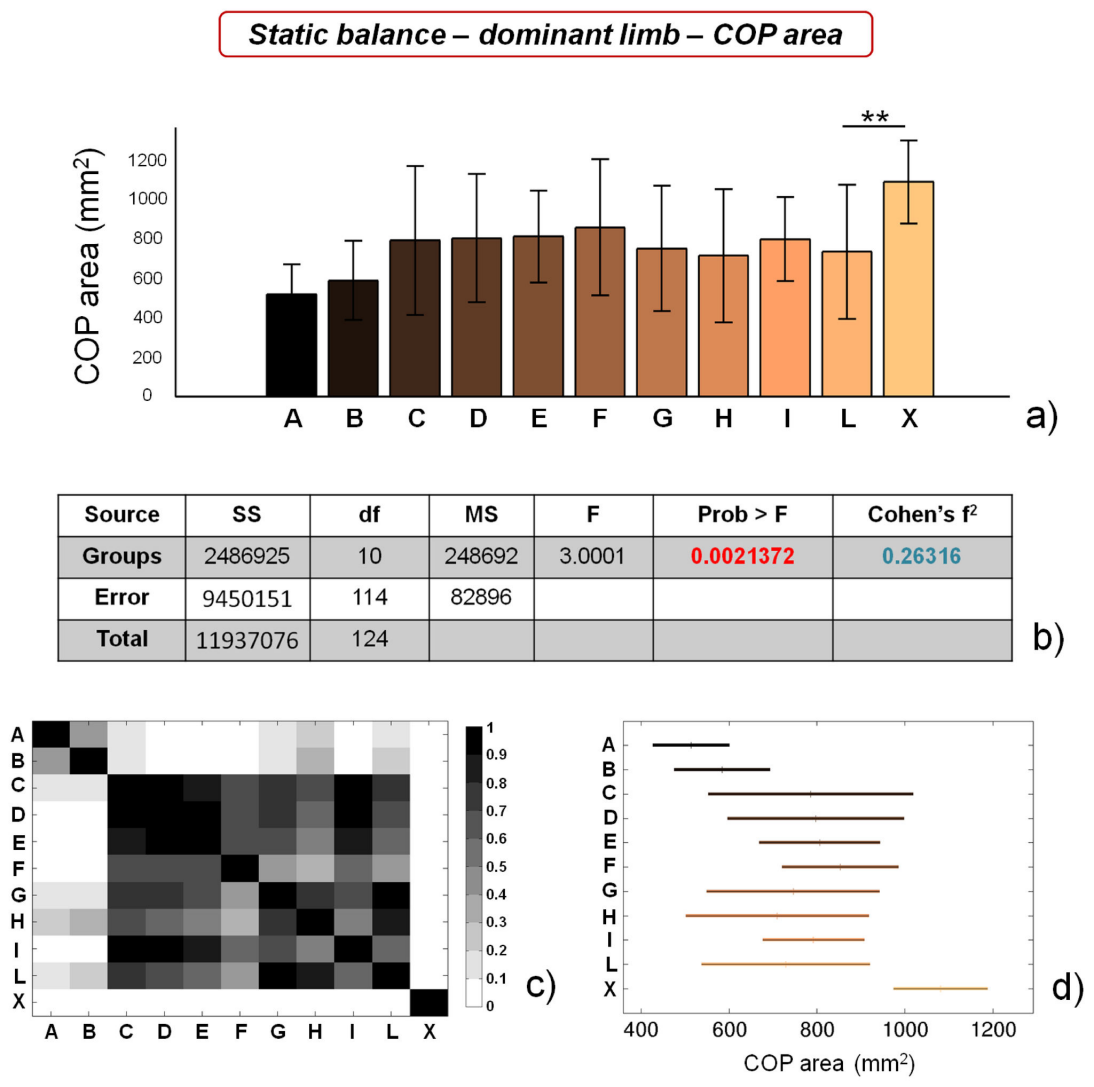
Results of static balance tests, evaluated by means of COP area values, on dominant limb. a) COP area values for the different analyzed groups (average value ± standard deviation), from A to X. **=p<0.01; b) results of the analysis of variance (ANOVA) and effect size (Cohen’s f^2^) calculation; c) matrix reporting the p values for the coupled t-tests between the different groups; d) multiple comparison plot (average values ± 95% of CI, with Bonferroni adjustement).

**Figure 6:**
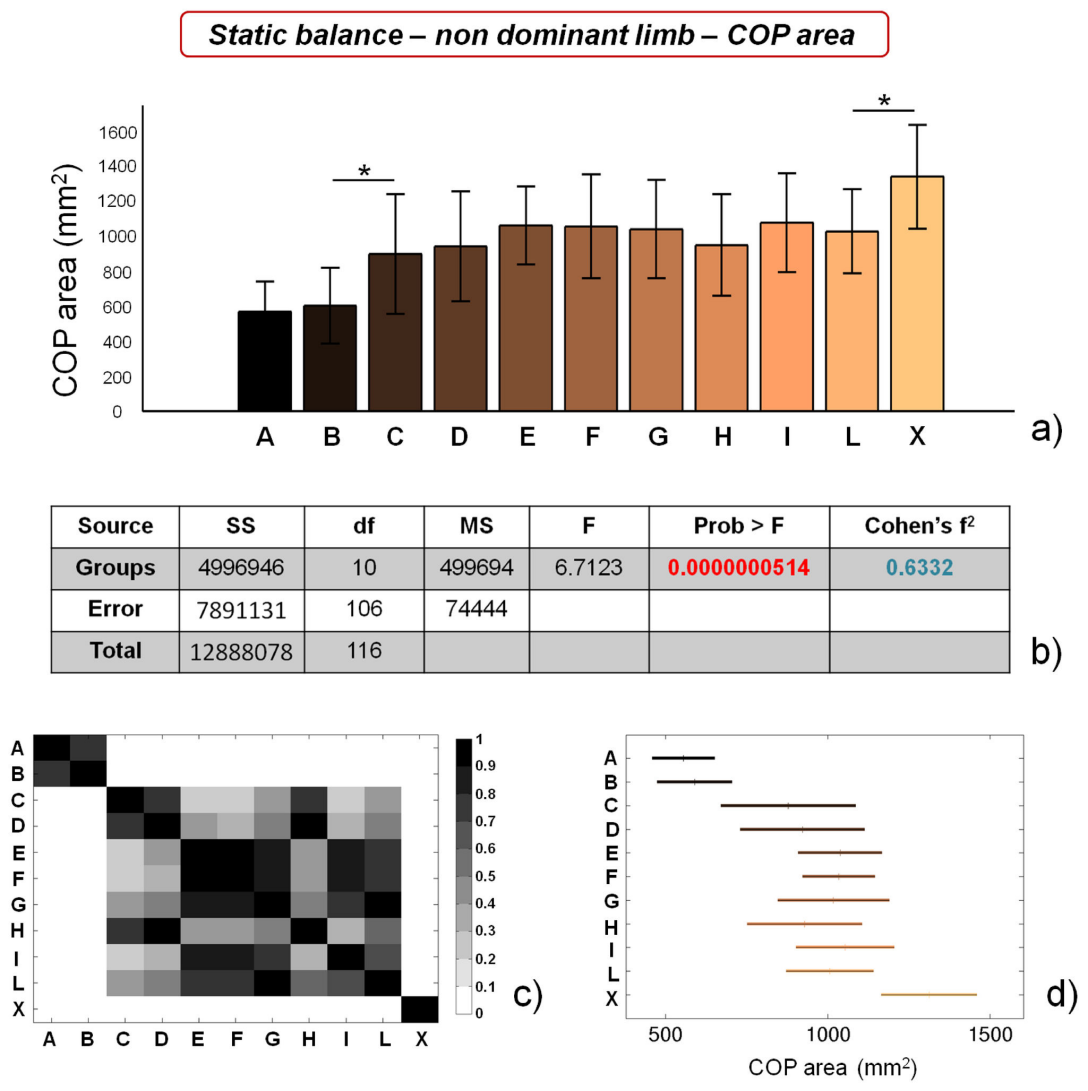
Results of static balance tests, evaluated by means of COP area values, on non dominant limb. a) COP area values for the different analyzed groups (average value ± standard deviation), from A to X. *=p<0.05; b) results of the analysis of variance (ANOVA) and effect size (Cohen’s f^2^) calculation; c) matrix reporting the p values for the coupled t-tests between the different groups; d) multiple comparison plot (average values ± 95% of CI, with Bonferroni adjustement).

COP area values evidence no significant differences between soccer players (groups from A to L) for dominant limb (Figure 5), while control subjects significantly differ (p<0.01) from soccer players belonging to group L. Significant differences can be found for the non dominant limb (Figure 6) between soccer players, which clearly identify two separate macro-groups. Furthermore, control subjects significantly differ (*=p<0.05) from soccer players belonging to group L. In the case of COP area values, the size of the macro-group of athletes showing high static balance performances is further reduced, in comparison with COP length values, being constituted by athletes playing only on A and B categories. ANOVA outcomes reveal that COP area values are statistically different for both dominant and non dominant limbs, though the difference is much higher in the case of non dominant limb. Cohen’s f^2^ effect size values also differ between dominant and non dominant limb, being ~ 0.26 (medium) in the first case and ~ 0.63 (high) in the second case.

Balance has been effectively used as predictor of injury risks [39,40] and proprioceptive training programs have been used to prevent lower limb injuries in many sports [41,42,43,44]. Furthermore, the reciprocal influence of balance and sport performance has been recently investigated. Concerning soccer, several studies reported that soccer training strongly influences balance abilities, especially concerning unilateral stance [45,46,47,48,49,50,51]. Conversely, it is also clear that intense balance training can improve some aspects of soccer performances, especially at early ages [52,53,54]. Recently, Paillard and colleagues focused on the analysis of postural performance and strategy of soccer players at different levels of competition. They found that national players have superior unipedal static balance than regional players [55].

Our results confirm the general insights already reported in literature, showing that high-level (professional) athletes are characterized by higher static balance performances in comparison with non-professional ones [19,55]. In addition, we were able to identify significant differences in static balance performance between professional athletes (showing the same training frequency): COP length values for both limbs were significantly different between group C and group D, while COP area values for non dominant limb were significantly different between group B and group C. These training-unrelated differences can be ascribed to intrinsic athlete’s abilities, such as greater sensitivity, a higher number of sensory receptors, better integration of information at the central nervous system level, more efficient afferent information at the vestibular or visual level, etc. 

Both COP length and COP area values did not significantly differ between dominant and non dominant limb, within the different categories, with the exception of categories E and I, which showed significantly smaller COP area values for non dominant limb (Table 2). These differences are probably due to the preferential use of non dominant limb, in soccer, for balancing the body during most technical movements (e.g. kicking, passing, etc.). However, this tendency is not confirmed for all the categories involved in the study.

**Table 2:** Comparison of COP length and COP area values (related to both static and dynamic balance tests) between dominant and non dominant (ND) limbs for athletes playing in the same categories or control subjects.

**Category subjected to comparisons**	**Static balance COP Length**		**Static balance COP Area**		**Dynamic balance COP Length - 3 s**		**Dynamic balance COP Length - 10 s**	
	Limb showing smaller values	p value	Limb showing smaller values	p value	Limb showing smaller values	p value	Limb showing smaller values	p value
A	-	0.33194	-	0.46389	-	0.05900	-	0.15000
B	-	0.23194	-	0.06597	-	0.63542	-	0.23056
C	-	0.61319	-	0.00417	-	0.43889	-	0.13125
D	-	0.56597	-	0.27014	-	0.58681	-	0.38681
E	-	0.42222	**ND**	**0.04600**	-	0.62292	-	0.36042
F	-	0.44375	-	0.06400	-	0.07361	-	0.22500
G	-	0.34444	-	0.00556	-	0.27639	-	0.26875
H	-	0.17083	-	0.11250	-	0.21875	**ND**	**0.01400**
I	-	0.65625	**ND**	**0.00139**	-	0.46736	-	0.00556
L	-	0.68889	-	0.07200	-	0.63333	-	0.25972	
X	-	0.52422	-	0.09244	-	0.13387	-	0.25223	

The limb showing smaller values is reported (if such differences were significant), together with the value of p for each comparison. If significant (p<0.05), p values are highlighted in bold.

### Dynamic balance performance

**Figure 7:**
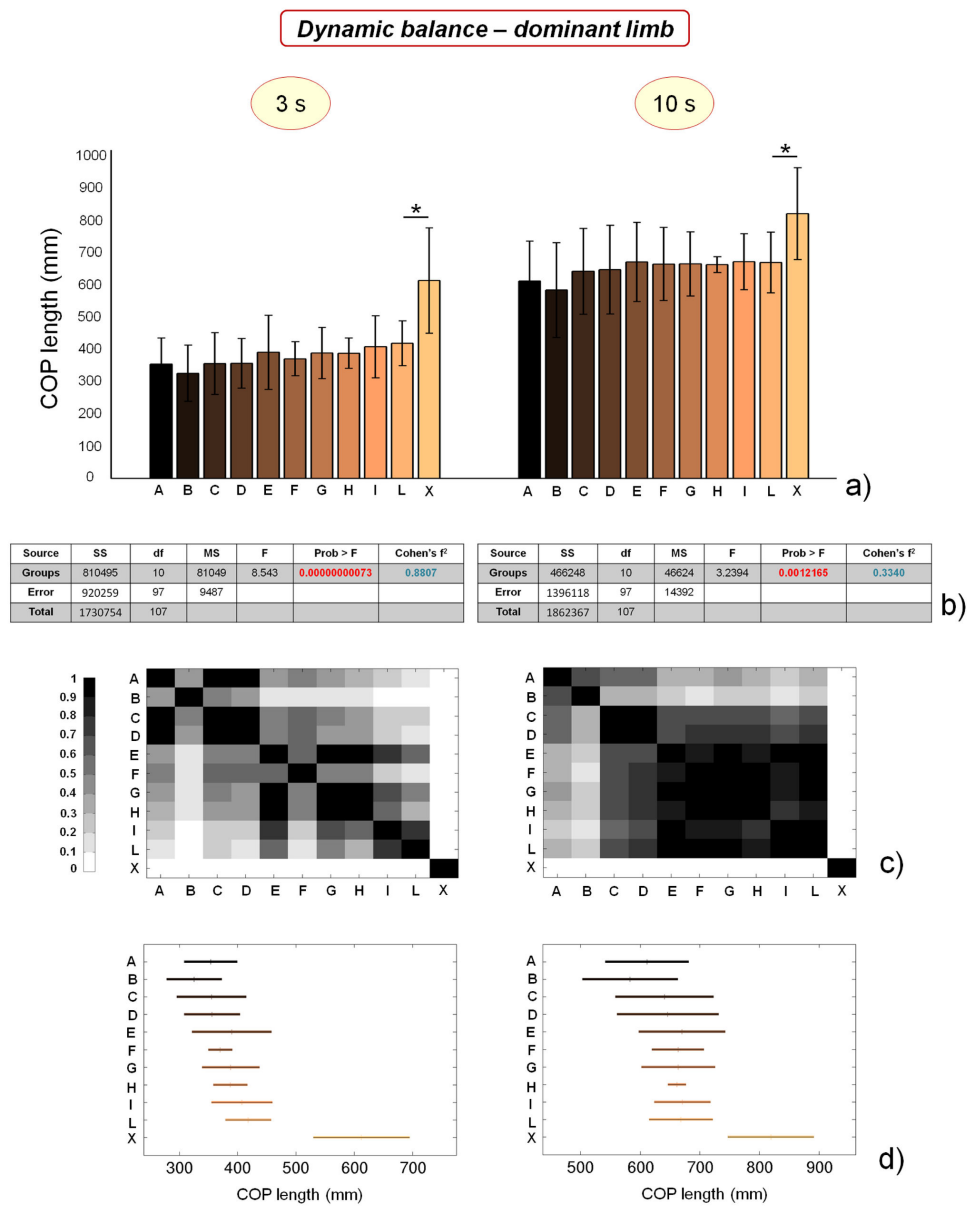
Results of dynamic balance tests, evaluated by means of COP length values calculated respectively 3 and 10 s after jump landing, on dominant limb. a) COP length values for the different analyzed groups (average value ± standard deviation), from A to X and for the different time-points. *=p<0.05; b) results of the analysis of variance (ANOVA) and effect size (Cohen’s f^2^) calculation for the different time-points; c) matrices reporting the p values for the coupled t-tests between the different groups, for the different time-points; d) multiple comparison plots (average values ± 95% of CI, with Bonferroni adjustement) for the different time-points.

Dynamic balance performance was evaluated by means of COP length values at respectively 3 and 10 s after landing on one foot from a jump task. These values provided information concerning the ability to recover a stable stance at different time-points. Figure 7 shows the results obtained for dominant limb. COP length values at 3 and 10 s appear significantly different (p<0.01 in both cases). However, as evidenced by Figure 7d, such difference is mainly due to control subjects, whose dynamic balance performances are greatly lower in comparison with those of soccer players. In fact, if we perform the ANOVA by excluding the control group X, we find no significant differences between the groups from A to L (p = 0.47 and 0.76 for COP length values after 3 s and 10 s, respectively). 

**Figure 8:**
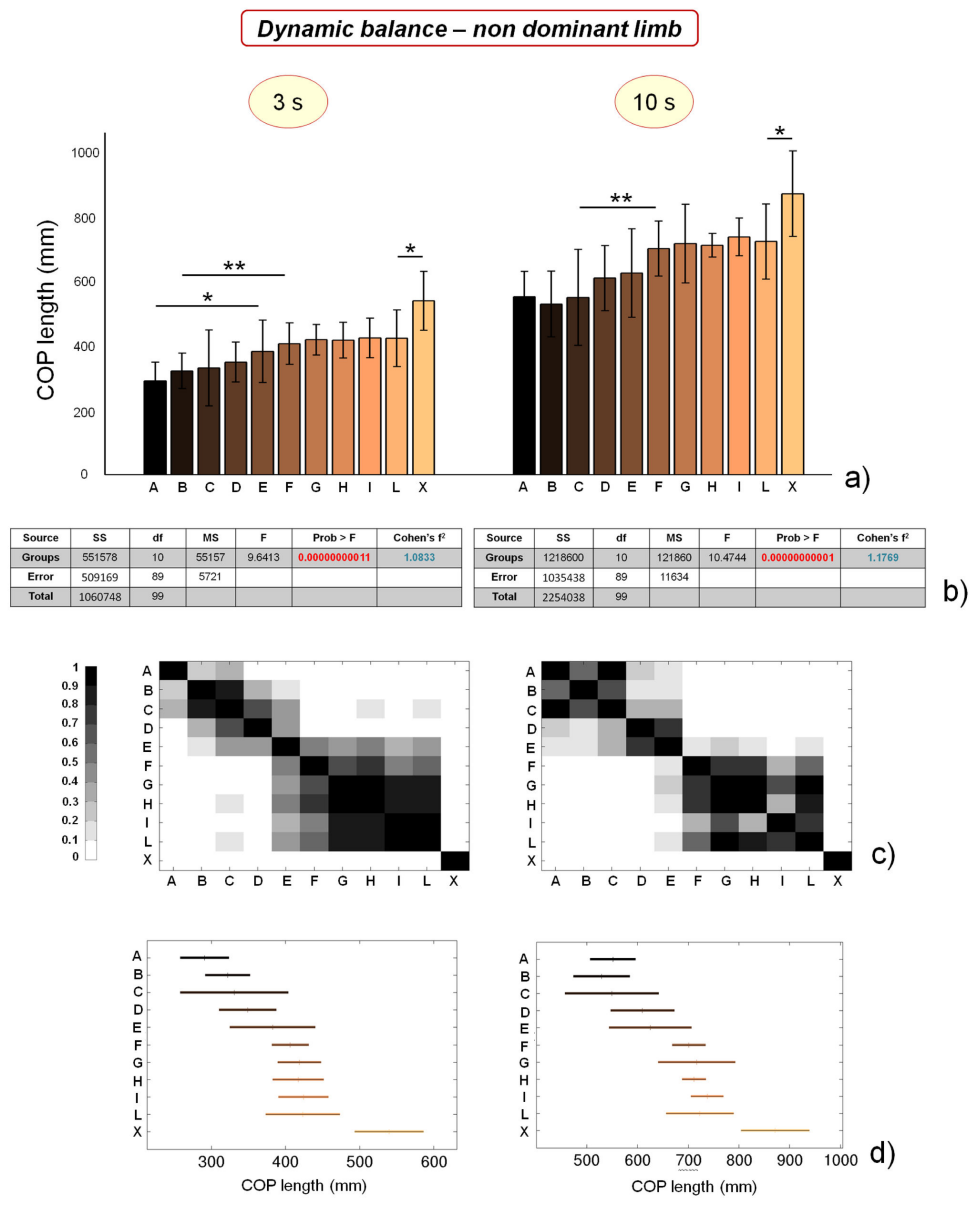
Results of dynamic balance tests, evaluated by means of COP length values calculated respectively 3 and 10 s after jump landing, on non dominant limb. a) COP length values for the different analyzed groups (average value ± standard deviation), from A to X and for the different time-points. *=p<0.05, **=p<0.01; b) results of the analysis of variance (ANOVA) and effect size (Cohen’s f^2^) calculation for the different time-points; c) matrices reporting the p values for the coupled t-tests between the different groups, for the different time-points; d) multiple comparison plots (average values ± 95% of CI, with Bonferroni adjustement) for the different time-points.

The same parameters, calculated for the non dominant limb, show different trends. Results, reported in Figure 8, show that dynamic COP length values are significantly different between the groups at both the time-points. Such differences are maintained (even if reduced) if we perform ANOVA by excluding the control group X: p values remain smaller than 0.01. Each parameter clearly identifies two macro-groups of athletes, almost corresponding to the division between professional and non professional soccer players. This conclusion is partly mitigated by the statistical analysis based on Bonferroni correction (Figure 8d), which does not confirm the relevant differences in athletes’ performance concerning dynamic balance on non dominant limb for the 3 s time-point. However, significant differences can be still found between athletes concerning COP length values 10 s after jump landing. Concerning data meta-analysis, Cohen’s f^2^ parameter values calculated for dynamic balance performances are larger in the case of non dominant limb, since significant differences can be found not only between soccer players and control subjects (as in the case of dominant limb), but also between professional and non professional soccer players.

The reasons of these differences in dynamic balance performances between professional and non-professional players can be found in the different strategy used to process postural-related information: it has been demonstrated that non-professional athletes use more short-loop information (proprioceptive myotactic and plantar cutaneous), while professional ones use more long-loop (vestibular) information [55].

Similarly to COP length and COP area values of static balance tests, dynamic balance results do not significantly differ between dominant and non dominant limb (the only significant difference concerns COP length values at 10 s for athletes playing in category H, see Table 2).

### Rapidity performance

**Figure 9:**
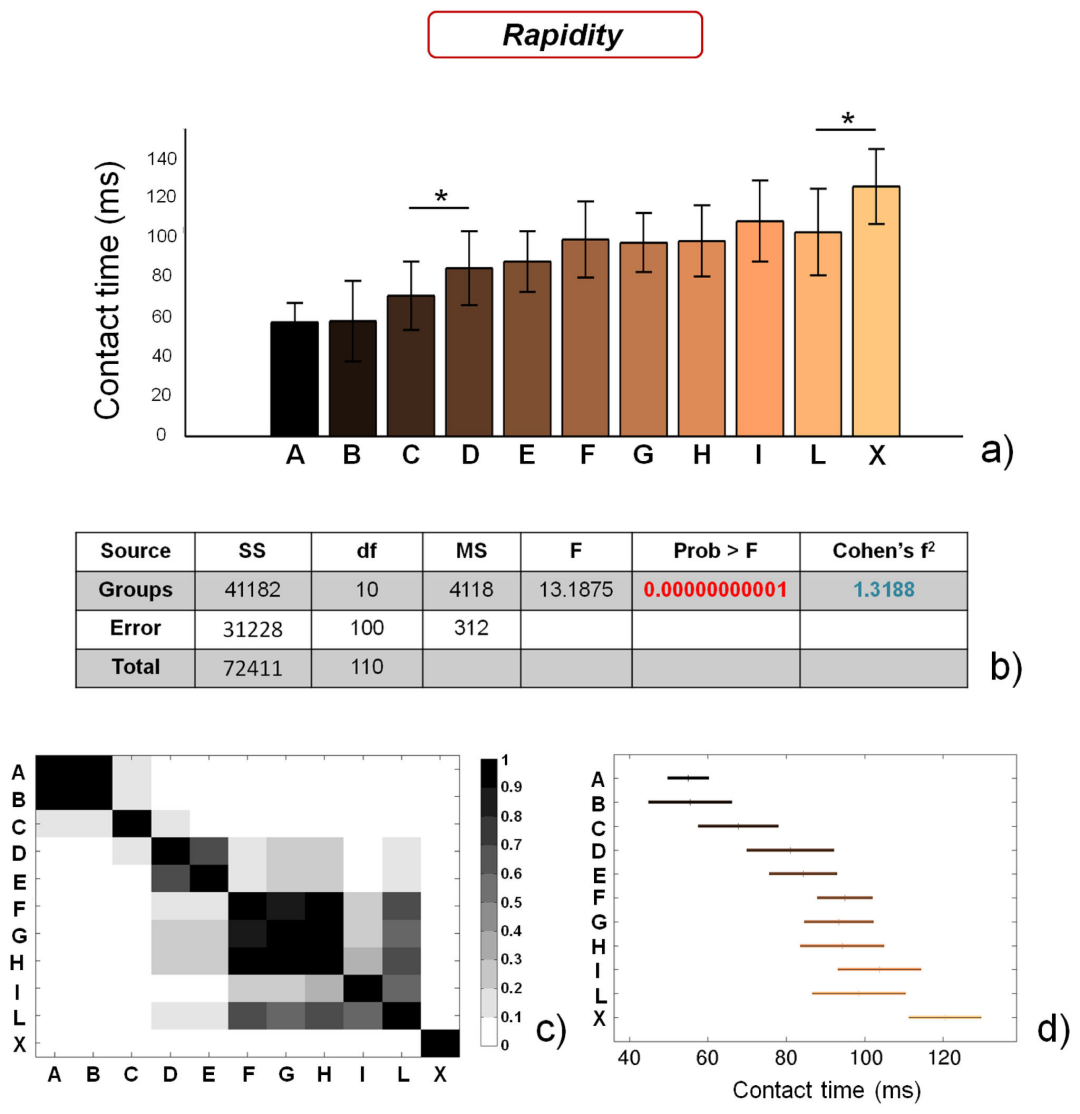
Results of the rapidity tests, evaluated by assessment of contact time values. a) contact time values for the different analyzed groups (average value ± standard deviation), from A to X. *=p<0.05; b) results of the analysis of variance (ANOVA) and effect size (Cohen’s f^2^) calculation; c) matrix reporting the p values for the coupled t-tests between the different groups; d) multiple comparison plot (average values ± 95% of CI, with Bonferroni adjustement).

Figure 9 reports the results obtained concerning subjects’ rapidity. Contact time values are significantly different between the groups (p < 0.01), with a large Cohen’s f^2^ (~ 1.32). Two macro-groups of soccer players can be identified, the former constituted by athletes playing in A, B and C categories, the latter constituted by athletes playing in categories from D to L. In addition, control subjects significantly differ (p<0.05) from athletes playing in the L category.

These results confirm the insights of recent studies [56,57] and highlight that only high-level athletes show significantly short contact values, Interestingly, we also found that some professional players strongly differ in terms of rapidity: the athletes of the A, B and C groups show significantly smaller contact times in comparison with those of the D group, thus highlighting that intrinsic factors (e.g. a higher nerve conduction velocity or a different muscle fibre composition) distinguish top-level players among those showing the same training frequency.

### Reaction times performance

**Figure 10:**
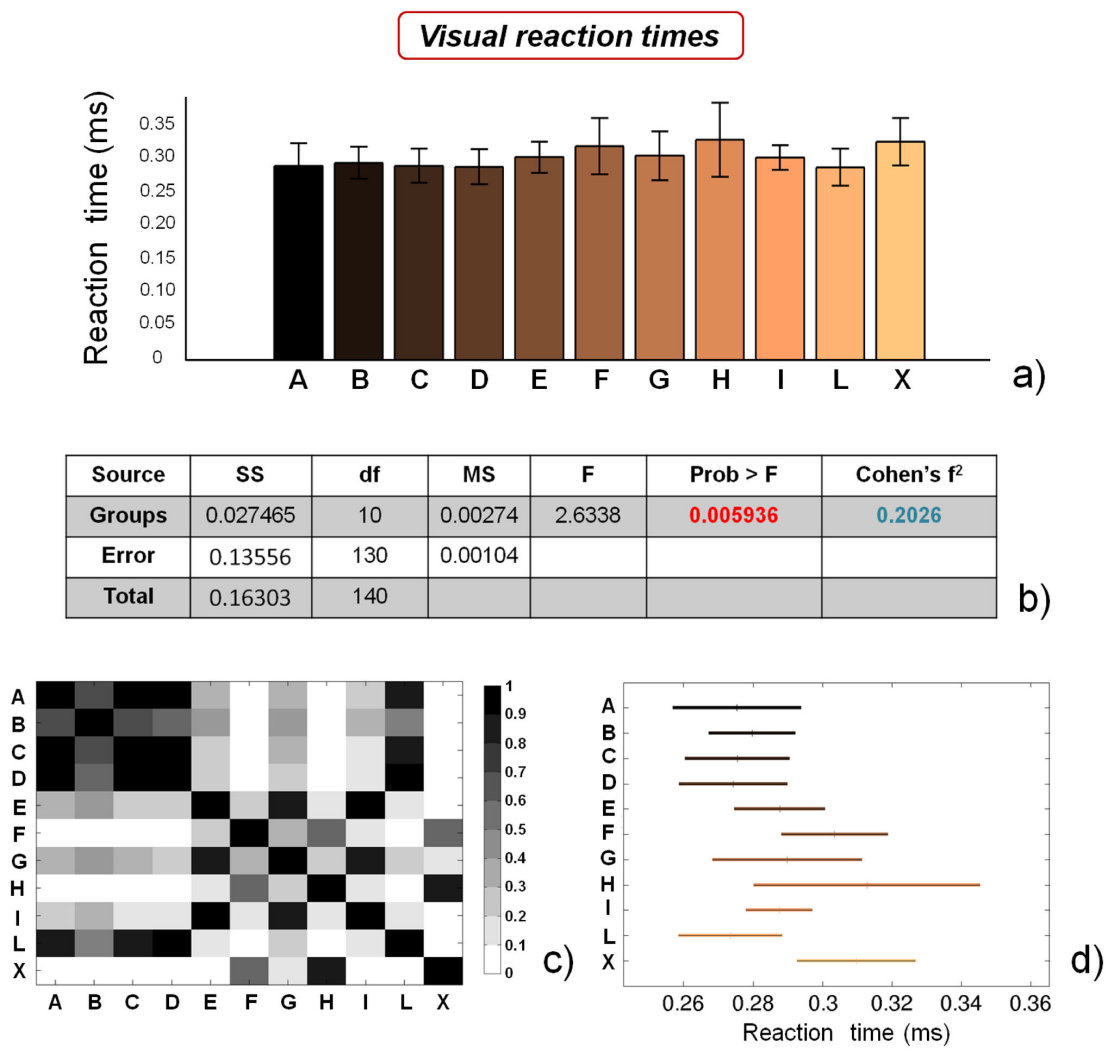
Results of the visual reaction times tests. a) reaction time values for the different analyzed groups (average value ± standard deviation), from A to X; b) results of the analysis of variance (ANOVA) and effect size (Cohen’s f^2^) calculation; c) matrix reporting the p values for the coupled t-tests between the different groups; d) multiple comparison plot (average values ± 95% of CI, with Bonferroni adjustement).

**Figure 11:**
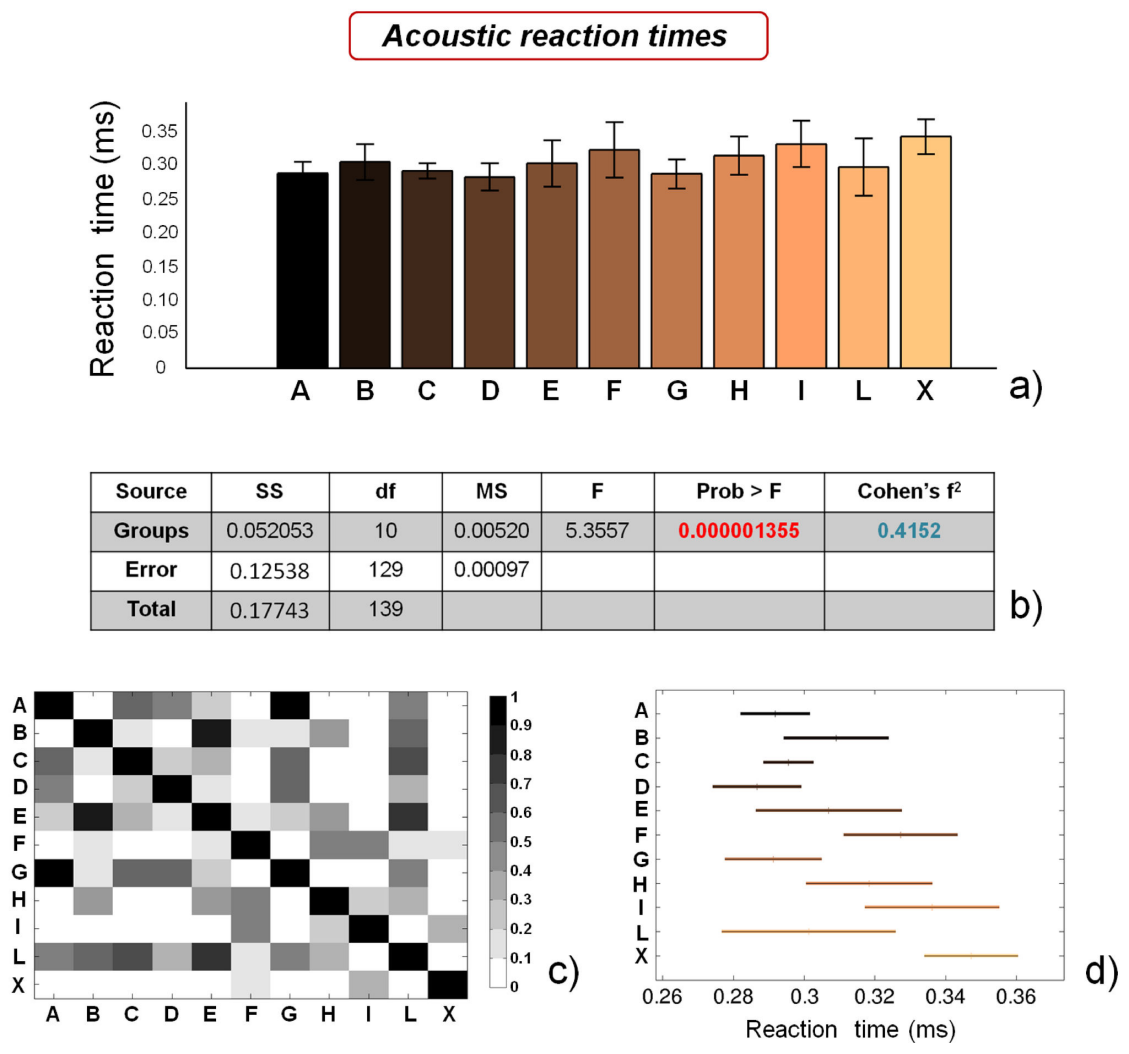
Results of the acoustic reaction times tests. a) reaction time values for the different analyzed groups (average value ± standard deviation), from A to X; b) results of the analysis of variance (ANOVA) and effect size (Cohen’s f^2^) calculation; c) matrix reporting the p values for the coupled t-tests between the different groups; d) multiple comparison plot (average values ± 95% of CI, with Bonferroni adjustement).

Figure 10 and Figure 11 report visual and acoustic reaction times, respectively. ANOVA results for both visual (Figure 10b) and acoustic (Figure 11b) reaction times highlight significant differences between the groups (p<0.01), while Cohen’s f^2^ values are ~ 0.20 (medium) and ~ 0.41 (large), respectively. However, the single comparisons between groups (Figure 10c,d and Figure 11c,d) highlight that no specific macro-groups can be identified, concerning reaction times. These insights are in contrast with recent findings [20,21], probably due to the small number of subjects analyzed in the mentioned studies.

### Non-parametric ANOVAs coupled with resampling method

**Figure 12:**
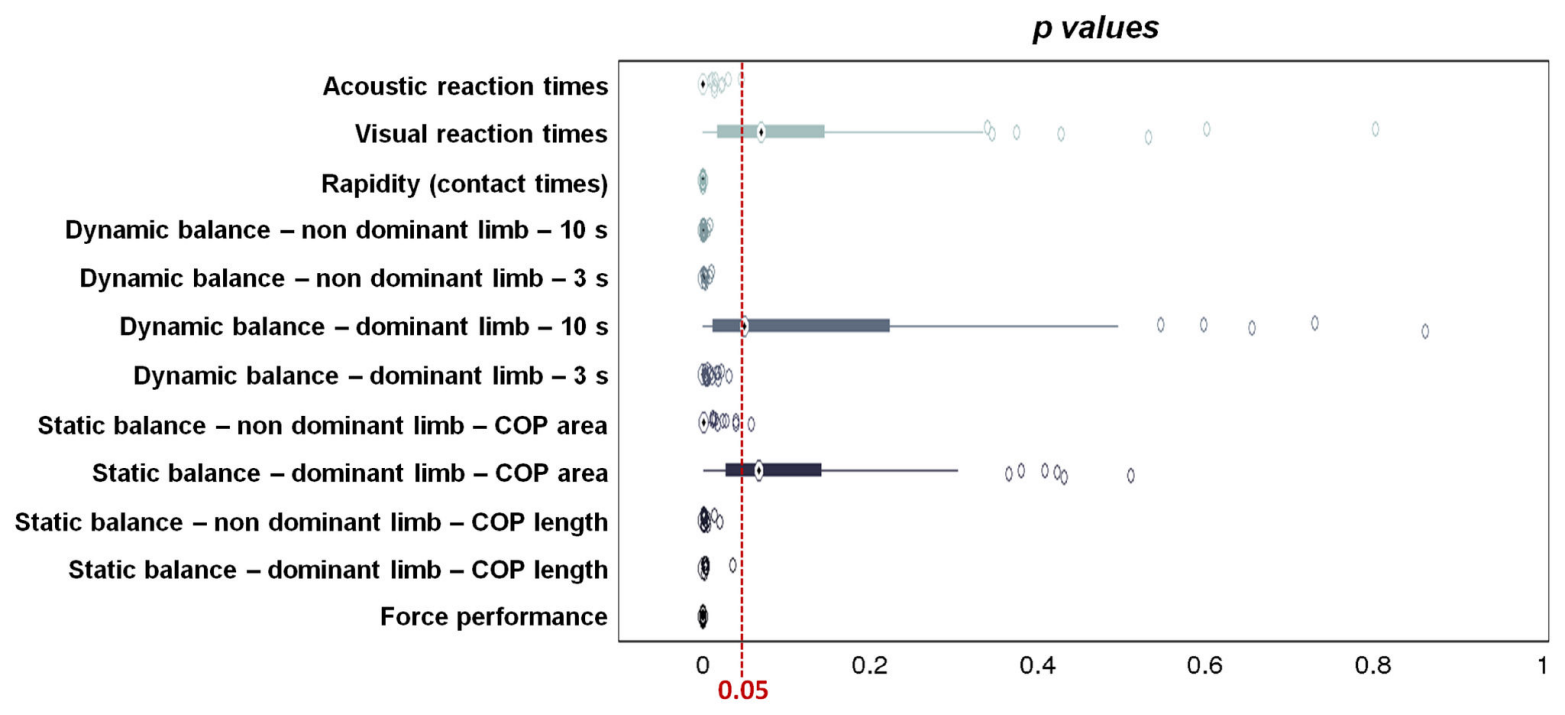
p values distributions for the different tests performed. 100 different p values were calculated for each test, by means of non-parametric ANOVAs, associated with a repeated random sub-sampling-based resampling method. Significance threshold was set at 0.05. Central boxes represent the central 50% of the data; their lower and upper boundary lines are the 25% / 75% quantile of the data. The two “whiskers” extend from the central box maximally to 1.5 times the length of the box. Points that remained out of this range (evidenced with empty circles) were considered as outliers.

Figure 12 shows the results (in terms of p value distributions) of 100 non-parametric ANOVAs performed for each test, by applying a repeated random sub-sampling-based resampling method, as described in the Materials and Methods section. Results show that p value distributions are well below the significance threshold (0.05) for all the performed tests, with exception of static balance (dominant limb, COP area), dynamic balance (dominant limb, 10 s) and visual reaction times. As previously reported, these tests were characterized by significant, but relatively high p values (0.002, 0.001 and 0.005, respectively), in comparison with ANOVA outcomes of the other tests. 

**Figure 13:**
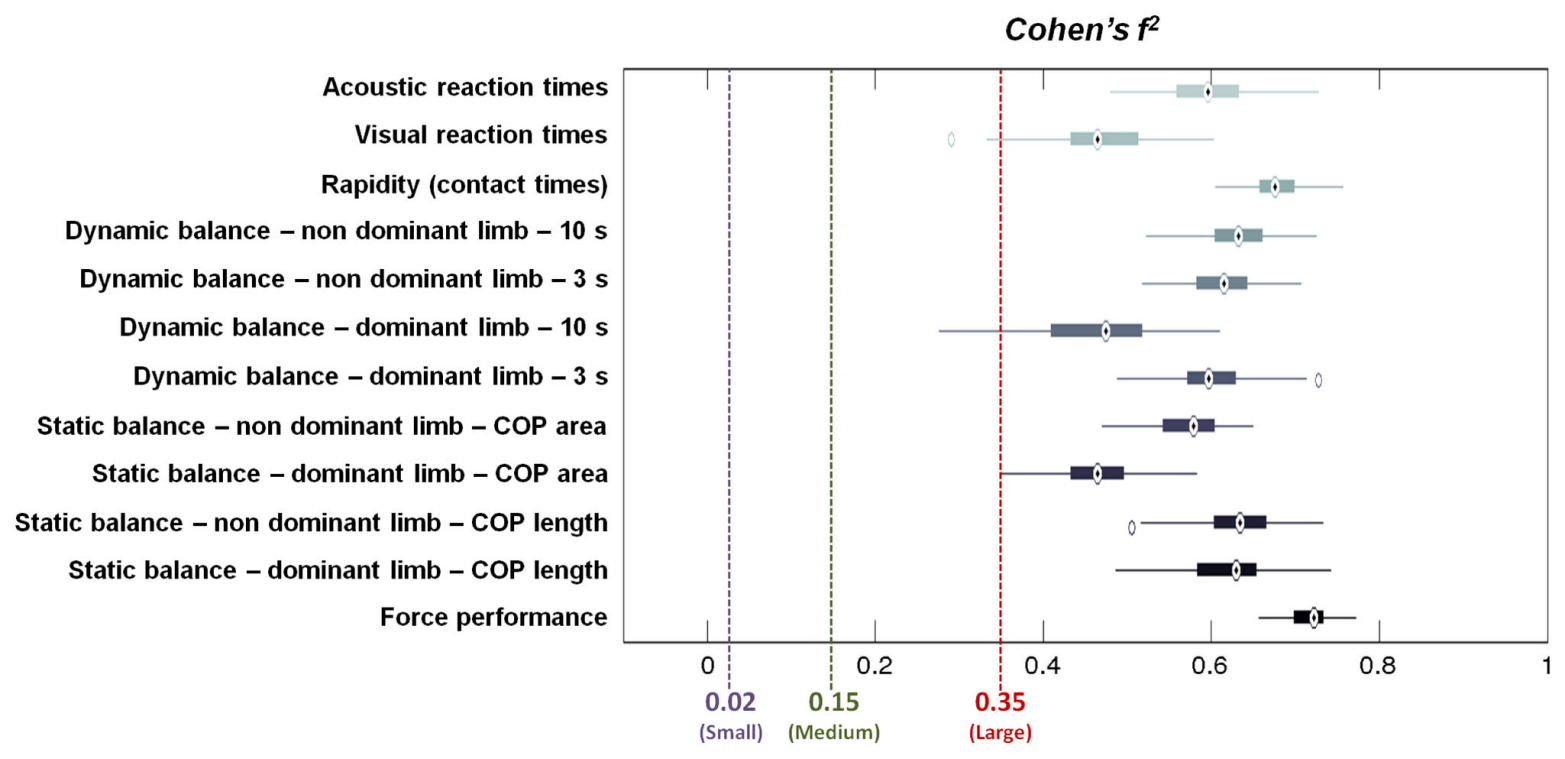
Cohen’s f^2^ values distributions for the different tests performed. 100 different f^2^ values were calculated for each test, by applying a repeated random sub-sampling-based resampling method. “Small”, “medium” and “large” thresholds were set at 0.02, 0.15 and 0.35, respectively. Central boxes represent the central 50% of the data; their lower and upper boundary lines are the 25% / 75% quantile of the data. The two “whiskers” extend from the central box maximally to 1.5 times the length of the box. Points that remained out of this range (evidenced with empty circles) were considered as outliers.

Figure 13 shows the Cohen’s f^2^ effect sizes distributions, calculated as described in (4), for the different tests performed. Results show that all the parameters are large (> 0.35), but the distributions correspondent to static balance (dominant limb, COP area), dynamic balance (dominant limb, 10 s) and visual reaction times are clearly characterized by smaller values. As previously reported, actually, these tests were characterized by Cohen’s f^2^ values (0.26, 0.33 and 0.20, respectively) which are much smaller than those of the other tests performed.

### PCA Analysis

**Figure 14:**
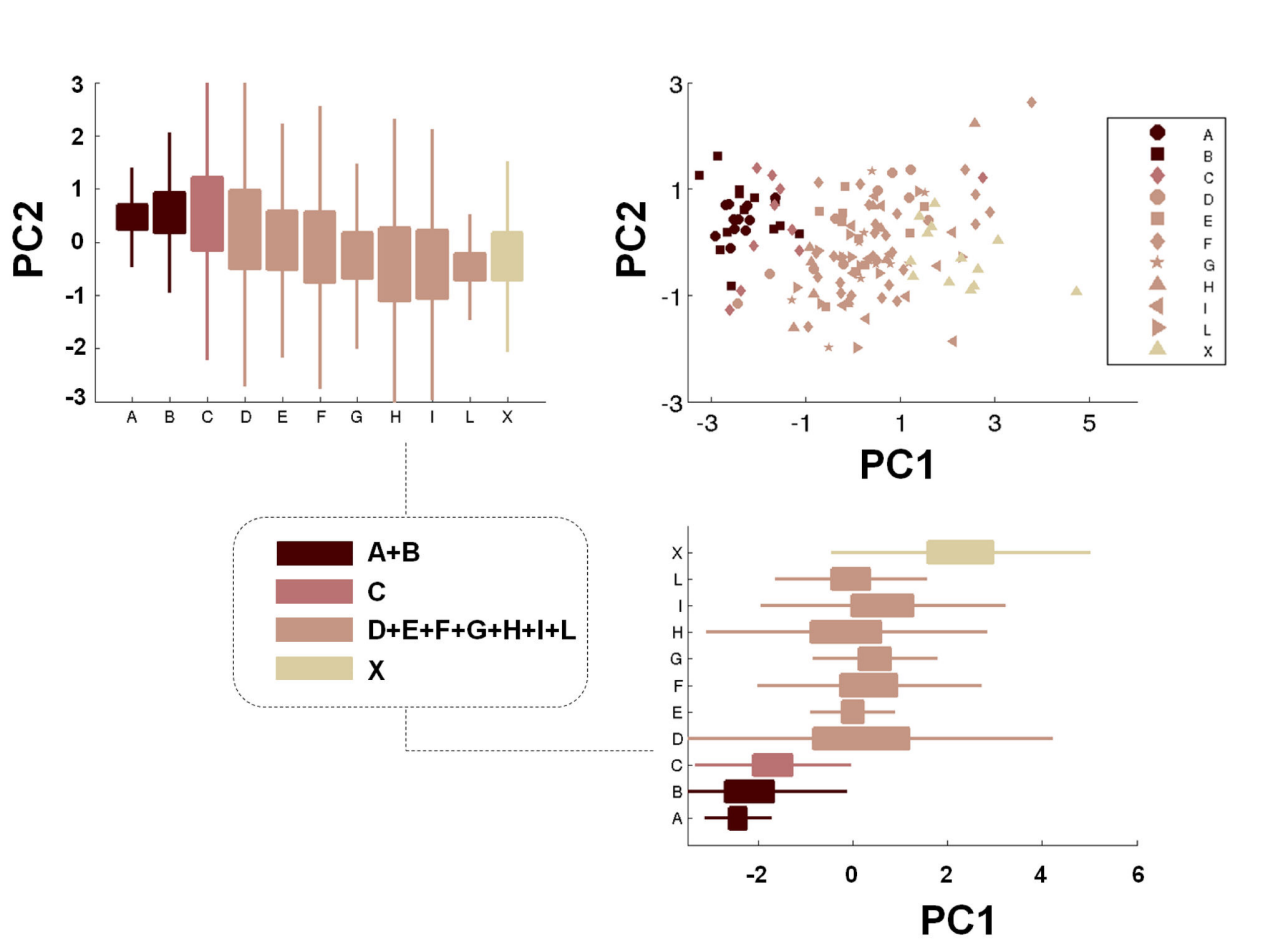
PCA analysis results. Scatter plot of the athletes playing in all the 10 analyzed categories and of control subjects on the principal component parameters plane and identification of four macro-groups for subjects’ clustering. PC1 and PC2 values for the different categories are also reported, by means of box-plots. Central boxes represent the central 50% of the data; their lower and upper boundary lines are the 25% / 75% quantile of the data. The two “whiskers” extend from the central box maximally to 1.5 times the length of the box. Points that remained out of this range were considered as outliers.

The PCA procedure was applied to the observations of the tests that highlighted larger differences between athletes playing in different categories, namely: force, static balance (COP length values for both dominant and non dominant limbs and COP area values, only concerning non dominant limb), dynamic balance (COP length values 10 s after landing, only for non dominant limb), and rapidity. Then, the first two PCs (PC1 and PC2), which were linear combinations of the six mentioned parameters and which accounted ~ 70% of the data variance, were used to scatter all the subjects, which were thus divided in four macro-groups: (i) the top-level divisions (A and B), (ii) a high-level division with the same training frequency of A and B, but probably including less talented players (C), (iii) the other lower-level divisions (D, E, F, G, H, I and L), which include players with markedly different training frequencies and (iv) the control group X. The results shown in Figure 14 highlight a clear separation of the average values between the four macro-groups in the PCA parameters space.

These results permit to define distinct regions of the PCA plane that correspond, according to our experimental data, to different groups of soccer categories or to non-athletes. This may serve as a reference to “characterize” players in terms of the six mentioned characteristics (checking in which region of the PCA plane they reside). Such characterization, of course, would not take into consideration technical nor tactical abilities, but it may help to identify the potential of certain players, and to timely train specific abilities at early ages, in order to exploit at the maximum level their specific age-related motor learning capability [53].

## Conclusions

170 soccer players from ten different Italian soccer leagues and 15 control subjects were analyzed in this study. Results revealed that force performance almost linearly decreases going from high-level to low-level athletes. Concerning static balance, COP length values for both limbs and COP area values for non dominant limb allow to identify two macro-groups of players, similarly to contact time values, representing acyclic rapidity performance. Dynamic balance on non dominant limb identifies two macro-groups of players almost reflecting the division between professional and non professional ones. Finally, both visual and acoustic reaction times do not discriminate soccer players at different levels of competition, or control (non-athletes) subjects. Six out of the twelve analyzed parameters, namely force, static balance (COP length values for both dominant and non dominant limbs and COP area values, only concerning non dominant limb), dynamic balance (COP length values 10 s after landing, only for non dominant limb), and rapidity permit to distinguish top-level athletes between players showing the same training frequency. PCA analysis allows the identification of four macro-groups of subjects showing similar key performances and evidences a specific portion of the principal component parameters plane corresponding to top-level athletes (those playing in A and B groups), thus highlighting the possibility of using the results of the present study to characterize and consequently train future athletes on the basis of their position on the PCA plane. 

## Supporting Information

File S1 **Experimental data.** The Microsoft Office Access Table contains the experimental data acquired during the study.
(MAT)

File S2 **Matlab code for Figures generation.** M file allowing the generation of Figures 2-14.
(M) Click here for additional data file.

File S3 **Matlab code for comparison D - ND.** M file allowing the generation of Table 2.
(M) Click here for additional data file.

File S4 **Matlab code for resampling.** M file allowing data resampling.
(M) Click here for additional data file.

File S5 **Matlab code for PCA.** M file dedicated to the Principal Component Analysis of experimental data.
(M) Click here for additional data file.

Movie S1 **Static and dynamic balance tests.** The movie shows a subject performing a static balance “single leg stance” task and a dynamic balance “jump and single leg stance” task on the sensorized platform.
(WMV)

Movie S2 **Rapidity test.** The movie shows a subject performing an “acyclic rapidity” test, by jumping on the sensorized platform and trying to minimize the contact time.
(WMV)

## References

[1] Dunning E (1999) Sports matters: sociological studies of sport, violence and civilization. New York: Taylor & Francis. 281 pp.

[2] FIFA (2007) Survey. 265 million playing football. FIFA Magazine: 10-15, Available: http://www.fifa.com/mm/document/fifafacts/bcoffsurv/emaga_9384_10704.pdf . Accessed 2013 Sept 11

[3] Weineck J (2004) Optimales training. Spitta Verlag GmbH & Co. 777 p.

[4] Elferink-Gemser MT, Visscher C, Lemmink KAPM, Mulder T (2007) Multidimensional performance characteristics and standard of performance in talented youth field hockey players: A longitudinal study. J Sports Sci 25: 481-489. doi:10.1080/02640410600719945. PubMed: 17365535. 17365535

[5] Rikberg A, Raudsepp L (2011) Multidimensional Performance Characteristics in Talented Male Youth Volleyball Players. Pediatr Exerc Sci 23: 537-548. PubMed: 22109779. 22109779 10.1123/pes.23.4.537

[6] Wisløff U, Castagna C, Helgerud J, Jones R, Hoff J (2004) Strong correlation of maximal squat strength with sprint performance and vertical jump height in elite soccer players. Br J Sports Med 38: 285-288. doi:10.1136/bjsm.2002.002071. PubMed: 15155427. 15155427 PMC1724821

[7] Wong DP, Chaouachi A, Dellal A, Smith AW (2012) Comparison of Ground Reaction Forces and Contact Times Between 2 Lateral Plyometric Exercises in Professional Soccer Players. Int J Sports Med 33: 647-653. doi:10.1055/s-0032-1304588. PubMed: 22510799. 22510799

[8] Winter DA, Patla AE, Frank JS (1990) Assessment of Balance Control in Humans. Med Prog Technol 16: 31-51. PubMed: 2138696. 2138696

[9] Grigg P (1994) Peripheral neural mechanisms in proprioception. J Sport Rehabil 3: 2-17.

[10] Nashner LM, Black FO, Wall C (1982) Adaptation to altered support and visual condition. J Neurosci 2: 536-544. PubMed: 6978930. 6978930 10.1523/JNEUROSCI.02-05-00536.1982 PMC6564270

[11] Palmieri RM, Ingersoll CD, Stone MB, Krause BA (2002) Center-of-pressure parameters used in the assessment of postural control. J Sport Rehabil 11: 51-66.

[12] Morasso PG, Spada G, Capra R (1999) Computing the COM from the COP in postural sway movements. Hum Mov Sci 18: 759-767. doi:10.1016/S0167-9457(99)00039-1.

[13] Karlsson A, Frykberg G (2000) Correlations between force plate measures for assessment of balance. Clin Biomech 15: 365-369. doi:10.1016/S0268-0033(99)00096-0. PubMed: 10758298. 10758298

[14] Hrysomallis C (2011) Balance Ability and Athletic Performance. Sports Med 41: 221-232. doi:10.2165/11538560-000000000-00000. PubMed: 21395364. 21395364

[15] Gerbino PG, Griffin ED, Zurakowski D (2007) Comparison of standing balance between female collegiate dancers and soccer players. Gait Posture 26: 501-507. doi:10.1016/j.gaitpost.2006.11.205. PubMed: 17197186. 17197186

[16] Ricotti L, Ravaschio A (2011) Break dance significantly increases static balance in 9 years-old soccer players. Gait Posture 33: 462-465. doi:10.1016/j.gaitpost.2010.12.026. PubMed: 21251832. 21251832

[17] Bressel E, Yonker JC, Kras J, Heath EM (2007) Comparison of static and dynamic balance in female collegiate soccer, basketball, and gymnastics athletes. J Athl Train 42: 42-46. PubMed: 17597942. 17597942 PMC1896078

[18] Paillard T, Noé F (2006) Effect of expertise and visual contribution on postural control in soccer. Scand J Med Sci Spor 16: 345-348. doi:10.1111/j.1600-0838.2005.00502.x. PubMed: 16978254. 16978254

[19] Ben Moussa AZ, Zouita S, Dziri C, Ben Salah FZ (2012) Postural control in Tunisian soccer players. Sci Sports 27: 54-56. doi:10.1016/j.scispo.2011.03.006.

[20] Spierer DK, Petersen RA, Duffy K (2011) Response Time to Stimuli in Division I Soccer Players. J Strength Cond Res 25: 1134-1141. doi:10.1519/JSC.0b013e3181d09e4c. PubMed: 20664362. 20664362

[21] Ruschel C, Haupenthal A, Hubert M, Fontana HB, Pereira SM et al. (2011) Simple reaction time in soccer players from differing categories and field positions. Motricidade 7: 73-82.

[22] Gribble PA, Tucker WS, White PA (2007) Time-of-day influences oft static and dynamic postural control. J Athl Train 42: 35-41. PubMed: 17597941. 17597941 PMC1896064

[23] Wilson G, Murphy A (1995) The efficacy of isokinetic, isometric and vertical jump tests in exercise science. Aust J Sci Med Sport 27: 20-24. doi:10.1249/00005768-199505001-00119. PubMed: 7780773. 7780773

[24] Lin D, Seol H, Nussbaum MA, Madigan ML (2008) Reliability of COP-based postural sway measures and age-related differences. Gait Posture 28: 337-342. doi:10.1016/j.gaitpost.2008.01.005. PubMed: 18316191. 18316191

[25] Geldhof E, Cardon G, De Bourdeaudhuij I, Danneels L, Coorevits P et al. (2006) Static and dynamic standing balance: test-retest reliability and reference values in 9 to 10 year old children. Eur J Pediatr 165: 779-786. doi:10.1007/s00431-006-0173-5. PubMed: 16738867. 16738867

[26] Swanenburg J, de Bruin ED, Favero K, Uebelhart D, Mulder T (2008) The reliability of postural balance measures in single and dual tasking in elderly fallers and non-fallers. BMC Muscoskelet Disord 9: 162. doi:10.1186/1471-2474-9-162. PubMed: 19068125. PMC2614424 19068125

[27] Weyand PG, Sternlight DB, Bellizzi MJ, Wright S (2000) Faster top running speeds are achieved with greater ground forces not more rapid leg movements. J Appl Physiol 89: 1991-1999. PubMed: 11053354. 11053354 10.1152/jappl.2000.89.5.1991

[28] Hamsher K, Benton AL (1977) The reliability of reaction time determinations. Cortex 13: 306-310. doi:10.1016/S0010-9452(77)80040-3. PubMed: 923269. 923269

[29] Benjamini Y (2010) Discovering the false discovery rate. J R Stat Soc B Stat Methodol 72: 405-416. doi:10.1111/j.1467-9868.2010.00746.x.

[30] Wold S, Esbensen K, Geladi P (1987) Principal Component Analysis 2. Chemometr Intell Lab. pp. 37-52.

[31] Cometti G, Maffiuletti NA, Pousson M, Chatard JC, Maffulli N (2001) Isokinetic strength and anaerobic power of elite, subelite and amateur French soccer players. Int J Sports Med 22: 45-51. doi:10.1055/s-2001-11331. PubMed: 11258641. 11258641

[32] Gissis I, Papadopoulos C, Kalapotharakos VI, Sotiropoulos A, Komsis G et al. (2006) Strength and speed characteristics of elite, subelite, and recreational young soccer players. Res Sports Med 14: 205-214. doi:10.1080/15438620600854769. PubMed: 16967772. 16967772

[33] Requena B, González-Badillo JJ, de Villareal ESS, Ereline J, García I et al. (2009) Functional Performance, Maximal Strength, and Power Characteristics in Isometric and Dynamic Actions of Lower Extremities in Soccer Players. J Strength Cond Res 23: 1391-1401. doi:10.1519/JSC.0b013e3181a4e88e. PubMed: 19620927. 19620927

[34] López-Segovia M, Marques MC, van den Tillaar R, González-Badillo JJ (2011) Relationships between vertical jump and full squat power outputs with sprint times in U21 soccer players. J Hum Kinet 30: 135-144. PubMed: 23487438. 23487438 10.2478/v10078-011-0081-2 PMC3588648

[35] Papaevangelou E, Metaxas T, Riganas C, Mandroukas A, Vamvakoudis E (2012) Evaluation of soccer performance in professional, semi-professional and amateur players of the same club. J Phys Educ Sport 12: 362-370.

[36] Rey E, Lago-Peñas C, Lago-Ballesteros J (2012) Tensiomyography of selected lower-limb muscles in professional soccer players. J Electromyogr Kinesiol, 22: 866–72. PubMed: 22776612. 22776612 10.1016/j.jelekin.2012.06.003

[37] Tillin NA, Jimenez-Reyes P, Pain MT, Folland JP (2010) Neuromuscular performance of explosive power athletes versus untrained individuals. Med Sci Sports Exerc 42: 781-790. doi:10.1249/MSS.0b013e3181be9c7e. PubMed: 19952835. 19952835

[38] Ryushi T, Hakkinen K, Kauhanen H, Komi P (1988) Muscle fiber characteristics, muscle cross-sectional area and force production in strength athletes, physically active males and females. Scand J Sports Sci 10: 7-15.

[39] McGuine TA, Greene JJ, Best T, Leverson G (2000) Balance as a predictor of ankle injuries in high school basketball players. Clin J Sport Med 10: 239-244. doi:10.1097/00042752-200010000-00003. PubMed: 11086748. 11086748

[40] Hrysomallis C (2007) Relationship between balance ability, training and sports injury risk. Sports Med 37: 547-556. doi:10.2165/00007256-200737060-00007. PubMed: 17503879. 17503879

[41] Söderman K, Werner S, Pietilä T, Engström B, Alfredson H (2000) Balance board training: prevention of traumatic injuries of the lower extremities in female soccer players? A prospective randomized intervention study. Knee Surg Sports Traumatol Arthrosc 8: 356-363. doi:10.1007/s001670000147. PubMed: 11147154. 11147154

[42] Malliou P, Gioftsidou A, Pafis G, Beneka A, Godolias G (2004) Proprioceptive training (balance exercises) reduces lower extremity injuries in young soccer players. J Back Musculoskelet Rehabil 17: 101-104.

[43] Kraemer R, Knobloch K (2009) A Soccer-Specific Balance Training Program for Hamstring Muscle and Patellar and Achilles Tendon Injuries: An Intervention Study in Premier League Female Soccer. Am J Sports Med 37: 1384-1393. doi:10.1177/0363546509333012. PubMed: 19567665. 19567665

[44] Gioftsidou A, Malliou P, Pafis G, Beneka A, Tsapralis K et al. (2012) Balance training programs for soccer injuries prevention. J Hum Sport Exerc 7: 639-647. doi:10.4100/jhse.2012.73.04.

[45] Gioftsidou A, Malliou P, Pafis G, Beneka A, Godolias G et al. (2006) The effects of soccer training and timing of balance training on balance ability. Eur J Appl Physiol 96: 659-664. doi:10.1007/s00421-005-0123-3. PubMed: 16416322. 16416322

[46] Thorpe JL, Ebersole KT (2008) Unilateral Balance Performance in Female Collegiate Soccer Athletes. J Strength Cond Res 22: 1429-1433. doi:10.1519/JSC.0b013e31818202db. PubMed: 18714247. 18714247

[47] Gstöttner M, Neher A, Scholtz A, Millonig M, Lembert S et al. (2009) Balance Ability and Muscle Response of the Preferred and Nonpreferred Leg in Soccer Players. Motor Control 13: 218-231. PubMed: 19454781. 19454781 10.1123/mcj.13.2.218

[48] Matsuda S, Demura S, Nagasawa Y (2010) Static One-Legged Balance in Soccer Players during Use of a Lifted Leg. Percept Mot Skills 111: 167-177. doi:10.2466/05.23.26.27.PMS.111.4.167-177. PubMed: 21058597. 21058597

[49] Bieć E, Kuczyński M (2010) Postural control in 13-year-old soccer players. Eur J Appl Physiol 110: 703-708. doi:10.1007/s00421-010-1551-2. PubMed: 20582432. 20582432 PMC2957582

[50] Jakobsen MD, Sundstrup E, Krustrup P, Aagaard P (2011) The effect of recreational soccer training and running on postural balance in untrained men. Eur J Appl Physiol 111: 521-530. doi:10.1007/s00421-010-1669-2. PubMed: 20924596. 20924596

[51] Teixeira LA, de Oliveira DL, Romano RG, Correa SC (2011) Leg Preference and Interlateral Asymmetry of Balance Stability in Soccer Players. Res Q Exerc Sport 82: 21-27. doi:10.1080/02701367.2011.10599718. PubMed: 21462682. 21462682

[52] Stray-Pedersen JI, Magnusen R, Kuffel E, Seiler S (2006) Sling Exercise Training Improves Balance, Kicking Velocity, and Torso Stabilisation Strength in Elite Soccer Players. Med Sci Sport Exer 38: S243. doi:10.1249/00005768-200605001-01069.

[53] Ricotti L (2011) Static and dynamic balance in young athletes. J Hum Sport Exerc 6: 616-628. doi:10.4100/jhse.2011.64.05.

[54] Bird SP, Stuart W (2012) Integrating Balance and Postural Stability Exercises into the Functional Warm-up for Youth Athletes. Strength Cond J 34: 73-79. doi:10.1519/SSC.0b013e31824f175e.

[55] Paillard T, Noé F, Rivière T, Marion V, Montoya R et al. (2006) Postural performance and strategy in the unipedal stance of soccer players at different levels of competition. J Athl Train 41: 172-176. PubMed: 16791302. 16791302 PMC1472651

[56] Yap CW, Brown LE (2000) Development of speed, agility, and quickness for the female soccer athlete. Strength Cond J 22: 9-12. doi:10.1519/1533-4295(2000)022.

[57] Jovanovic M, Sporis G, Omrcen D, Fiorentini F (2011) Effects of Speed, Agility, Quickness Training Method on Power Performance in Elite Soccer Players. J Strength Cond Res 25: 1285-1292. doi:10.1519/JSC.0b013e3181d67c65. PubMed: 21522073. 21522073

